# Complete Genome Sequences of Six Chi-Like Bacteriophages That Infect Proteus and Klebsiella

**DOI:** 10.1128/mra.01215-21

**Published:** 2022-03-17

**Authors:** Hunter K. Cobbley, Seth I. Evans, Hannah M. F. Brown, Braden Eberhard, Nathaniel Eberhard, Minji Kim, Haley Mickelsen Moe, Daniel Schaeffer, Ruchira Sharma, Daniel W. Thompson, Sherwood R. Casjens, Julianne H. Grose

**Affiliations:** a Department of Microbiology and Molecular Biology, Brigham Young University, Provo, Utah, USA; b School of Biological Sciences, University of Utah, Salt Lake City, Utah, USA; c Division of Microbiology and Immunology, Department of Pathology, University of Utah School of Medicine, University of Utah, Salt Lake City, Utah, USA; DOE Joint Genome Institute

## Abstract

Proteus mirabilis and Klebsiella aerogenes are Gram-negative opportunistic pathogens that are responsible for nosocomial and health care-associated infections, including urinary tract infections. Here, the full genome sequences of six Chi-like Proteus (DanisaurMW, DoubleBarrel, Inception, Jing313, and NotEvenPhaged) or Klebsiella (Phraden) bacteriophages are announced, contributing to the understanding of Chi-like phages.

## ANNOUNCEMENT

There are currently over 50 genomes of Chi-like phages that primarily infect Salmonella in GenBank; however, Chi-like phages that infect Escherichia, *Cronobacter*, Proteus, *Providencia*, Klebsiella, *Erwinia*, *Serratia*, and Enterobacter are also known. Here, six Chi-like bacteriophages were isolated from a 37°C LB enrichment culture containing raw sewage from local wastewater treatment plants and Proteus mirabilis Hauser ATCC 7002 or Klebsiella aerogenes ATCC 13047, expanding our understanding of this phage family. Enrichment cultures were plated in LB top agar, and single plaques were picked and subsequently purified through at least three successive rounds of single plaque isolation. These bacteriophages were grown in liquid cultures and centrifuged to remove cells and debris, and the resulting high-titer lysates (>10^8^) were used to extract DNA with a phage DNA isolation kit (Norgen Biotek, Canada). Genomic DNA was prepared for 150-bp paired-end Illumina iSeq sequencing (Brigham Young University) using the NEBNext Ultra II DNA library preparation kit for all phages except Phraden, whose genome was prepared using the Illumina TruSeq Nano DNA library preparation kit, followed by 150-bp paired-end MiSeq sequencing (University of Utah). All contigs were assembled and trimmed using the preset *de novo* assembly function of Geneious v.8.0. 5 (1) except for Phraden’s genome, for which v.R11 was used, and genomes were annotated using DNA Master (2) and GeneMarkS (3). All software was used with default settings.

All six phages showed overall homology (∼60% identity over 50% of the genome) to the Salmonella phages of the previously described *Enterobacteriales* Chi-like phage cluster (4) known as the *Chivirus* genus. Jing313 circularized upon assembly, but all other phages were linear and thus consistent with Chi-like cohesive end packaging mechanisms (5). PhageTerm (6) was used to analyze raw sequence reads and predicted cohesive ends for phage Jing313, consistent with the 12-base single-stranded DNA extensions with the sequence 5′-GGTGCGCAGAGC (the same as in Chi [5]). The same ends were present in the sequences of all six phage genomes.

The genome sizes of the six phages reported here are typical of Chi-like phages (∼58,000 to 60,000 bp [4]), but their G+C contents vary with the host (Table 1); the Proteus phage genomes sequenced here (DanisaurMW, Inception, Jing313, NotEvenPhaged, and DoubleBarrel) have G+C contents of ∼46.8%, and the G+C content of the Klebsiella phage (Phraden) genome is 56.6%. These G+C contents are consistent with the lower G+C content reported for Proteus mirabilis (∼39% [7, 8]) and the higher G+C content for Klebsiella aerogenes (∼55% [9]).

**TABLE 1:** Sequencing summary and basic properties of six Chi-like *Enterobacteriales* phages

Phage name^*a*^	GenBank accession no.	SRA accession no.	Total no. of reads	Sequencing coverage (range [mean]) (×)	Length (bp)	G+C content (%)	Sewage sampling location coordinates
vB_PmiS_DanisaurMW	OK499998	SRR17231388	284,602	404–989 (732.6)	58,538	46.9	41.1324°N, 111.9302°W
vB_PmiS_DoubleBarrel	OK500000	SRR17231358	59,077	7–253 (110.2)	59,089	46.8	40.2969°N, 111.6946°W
vB_PmiS_Inception	OK499974	SRR17231379	213,515	246–726 (549.1)	58,589	46.8	40.8894°N, 111.8808°W
vB_PmiS_Jing313	OK499975	SRR17231363	203,184	143–734 (523.4)	58,534	46.9	40.2338°N, 111.6585°W
vB_PmiS_NotEvenPhaged	OK499986	SRR17231368	140,894	1–519 (360)	58,566	46.9	40.7608°N, 111.8910°W
vB_KaeS_Phraden	OL606627	SRR17231385	43,212	1,855–4,720 (2,871.1)	59,034	56.6	34.0551°N, 117.7500°W

a Phage names contain host information, i.e., vB_Pmi for Proteus mirabilis phage and vB_Kae for Klebsiella aerogenes phage.

The Salmonella Chi-like phages that have been studied utilize the host’s actively rotating flagellum as a receptor (10–15). In phage YSD1, genes at the transcriptionally downstream end of the tail gene cluster are thought to encode the long curly tail tip fiber that likely mediates this process (11), and all six phages reported here contain homologues of these genes; therefore, it is likely that these Chi-like phages that infect Proteus and Klebsiella also utilize flagellum receptors. The P. mirabilis and K. aerogenes phages reported here will broaden our understanding of this common phage group.

### Data availability.

The accession numbers for all six bacteriophage genomes sequenced here are found in Table 1.
